# Positive Impact of Targeted Educational Intervention in Children With Low Adherence to Growth Hormone Treatment Identified by Use of the Easypod™ Electronic Auto-injector Device

**DOI:** 10.3389/fmedt.2021.609878

**Published:** 2021-03-15

**Authors:** Aria Reza Assefi, Fernanda Roca, Adrián Rubstein, Cinthia Chareca

**Affiliations:** ^1^Medical Department, Merck S.A., Buenos Aires, Argentina, an affiliate of Merck KGaA, Darmstadt, Germany; ^2^Patient Support Program, Medical Department, Merck S.A., Buenos Aires, Argentina, an affiliate of Merck KGaA, Darmstadt, Germany

**Keywords:** compliance, educational intervention, eHealth, growth disorders, growth hormone, medication adherence

## Abstract

**Background:** It is important to identify patients with low adherence to recombinant human growth hormone (r-hGH) therapy and initiate actions to improve adherence. The Merck Patient Support Program (PSP) aims to raise the awareness of these patients and their parents of the importance of good adherence in achieving optimal growth outcomes. The easypod™ digitally-enhanced injection device provides accurate, reliable adherence data for the PSP by recording the exact dose, time and date of injections given. In this study, we aimed to measure the effect of an educational intervention on adherence in patients using the easypod™ device to deliver their r-GH therapy.

**Methods:** This was a 12-month observational, retrospective cohort study. Patients previously identified by data recorded from their easypod™ injection device as having low adherence (<80%) were followed over the 6 months before and after a targeted educational visit by a PSP nurse. Patient adherence and demographic data were extracted from the PSP database. Statistical analyzes were carried out with STATA 15.0 software.

**Results:** Data from 80 patients (65% male) with low adherence were analyzed. Patients were aged 2–18 (mean: 11.77) years with diagnoses of growth hormone deficiency (71.25%), small for gestational age (20%), Turner syndrome (7.50%) and chronic renal disease (1.25%). Duration of treatment was 0.40–11.13 (median: 3.62) years. At baseline, median adherence to r-hGH therapy was 67%; after the intervention it increased to 76%, a statistically significant median improvement of 9% (*p* = 0.0000, Wilcoxon signed-rank test). Additionally, 36% (29/80) of patients increased their adherence to r-hGH therapy to ‘good’ (≥80%). Both changes were clinically relevant.

**Conclusions:** We conclude that a nurse-led educational intervention, supported by digital medication adherence monitoring, is a simple method to improve adherence to r-hGH therapy, and recommend this intervention to reduce the gap between the indication/recommendation of the specialist and patients' behavior.

## Introduction

The success of treatment for any chronic disease depends not just on the effectiveness of the medication but also on the patient's adherence to the treatment regimen over the long periods involved (1, 2). Poor adherence to long-term treatment is common, averaging 50% even in developed countries (1) and may be intentional or unintentional in origin. It can be due to fear of side-effects, forgetfulness, erratic lifestyle, variations in the severity of the disease itself, the complexity of the treatment regimen, or the perceived unpleasantness of the method for taking the medication, particularly when this involves injections (1). Other factors include age, adolescence, duration of treatment, relationship with the physician, cost and ease of access to medication and cognitive function (Table 1) (1, 3).

**Table 1:** Factors that can contribute to therapeutic non-adherence in patients treated with growth hormone (1, 3).

**Factor**
• Doctor–patient relationship
• Chronic and “silent” disease
• Lack of perceived effect of medication
• Complex injection process
• Pain during injection
• Fear of the use of needles
• Fear of side-effects
• Inadequate family support in children
• Tolerability problems
• Cognitive impairment
• Forgetfulness
• Patient knowledge and beliefs
• Cost of medication

According to the World Health Organization (WHO), adherence is a complex issue involving not only a person's behavior in relation to taking their medication as prescribed but also implementing changes in their lifestyle in response to the recommendations of their physician (1). A common definition of good adherence is taking ≥80% of the number of doses prescribed by the physician over the relevant time period (4).

In the specific case of growth disorders or growth hormone deficiency (GHD), a range of different factors can result in a low adherence rate to recombinant human growth hormone (r-hGH) therapy. This treatment requires a daily subcutaneous injection over a period of several years, and is most often required by very young children (5, 6). The condition being treated does not pose a threat to the patient's life or overall health and the benefits of the treatment are not seen immediately, and this can reduce the motivation needed to comply fully with the therapeutic regimen by the patients or their parents or carers (6, 7). Daily injections may be seen as burdensome by both the young patient and their family (7, 8) and, in many countries, the cost of r-hGH treatment and access to treatment are barriers to full adherence (9).

As a result, clinical studies have reported that failure to adhere to the treatment regimen is common in patients needing treatment with r-hGH and is associated with worse clinical and growth outcomes, along with economic inefficiency (9). Measurement of adherence to treatment has previously only been possible by proxy methods, based on estimates from questioning and observation of patients, medication diaries, or monitoring of prescriptions filled or vials returned (1, 9, 10). These different methodologies explain the variable nature of available data on adherence to treatment with r-hGH (7, 9). Studies that have assessed therapeutic adherence indirectly, through questionnaires addressed to patients or parents, report that between 48.0 and 78.7% of patients have “good” adherence to the injection of r-hGH (7). However, it is important to note that this measurement method depends on the reliability of the patient/parent and their recall, which frequently results in an overestimation of adherence (7, 11).

Nowadays, however, it is possible to obtain reliable, accurate, and objective data on therapeutic adherence through use of an electronic monitoring device, supported by observation of biochemical markers of response (10). The easypod™ is a digitally enhanced electromechanical injection device that enables accurate, objective measurement of adherence as it records the exact dose, time, and date of the injections made for each individual patient (12). This enables the physician to monitor adherence over time and, where necessary, design strategies to improve adherence in patients observed to have low adherence. A number of previous studies have shown that use of this device helps patients to maintain high levels of adherence over periods as long as 4 to 5 years (7, 13). The easypod™ device is part of an eHealth ecosystem, in which data from the device can be transmitted by the patient *via* the connect software to the patient's physician and also anonymized and saved to a database on a secure server for further analysis.

Patient support programs (PSPs) can provide additional support for patients and their carers in understanding their disease and responding appropriately to the demands of their treatment regimen. Merck Argentina has a PSP specifically designed to help patients receiving r-hGH treatment to improve the quality of their treatment and outcomes. The Merck PSP has two teams: one working from an operator call center that typically contacts the patient/parent every 3 months; the other comprises a PSP nurse service that makes routine 6-monthly visits to the patient's home. Call center staff also send device-related supporting material to patients.

The additional PSP-instigated, nurse-led educational interventions described in this study were specifically aimed at helping patients with low adherence, and their parents, to understand the importance of good adherence to treatment in achieving growth and clinical outcomes. We conducted this study to directly measure the effect of these interventions on patient adherence.

## Materials and Methods

**Figure 1:**
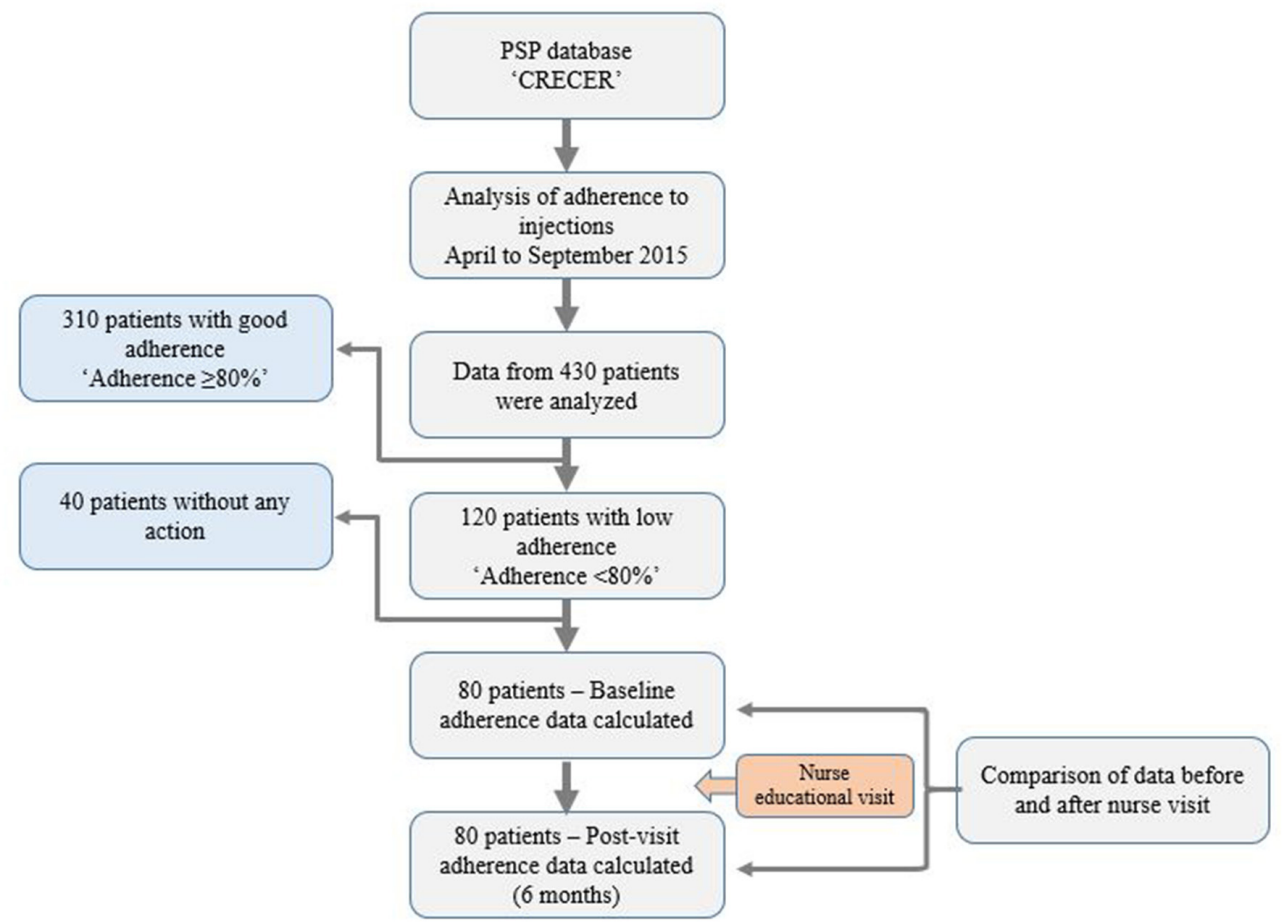
CONSORT style flow chart of study procedures.

This was an observational, retrospective analysis involving data from 430 patients (Figure 1). The data were collected from the PSP known as “CRECER,” meaning “growing up,” for a 6-month period from April 2015 to September 2015. Patients were classified according to their easypod™-recorded level of adherence to treatment for each complete week during this 6-month period, as follows:

- Good adherence (proportion of days with injection received/days with injections planned per week) was defined as ≥80% (*n* = 310).
- Low adherence (proportion of days with injection received/days with injections planned per week) was defined as <80% (*n* = 120).

All patients receiving Saizen® r-hGH (somatropin, Merck KGaA, Darmstadt, Germany) *via* easypod™ in Argentina are routinely visited by their personally assigned nurse (one of a team of six) who provides general educational advice about the correct use and storage of the products and devices required for their treatment and about improving the patient's quality of life; for example, by making dietary changes, ensuring good personal hygiene and a discussion of the patient's recreational, physical and cognitive activities. These educational visits also aim to improve a patient's adherence to their r-hGH treatment.

By discussing the adherence data recorded by the easypod™ device with their patients, nurses can investigate the possible causes of low adherence and, subsequently, design personalized action plans to improve this. These may include adapting injection routines for individual patients, providing reminders to patients to administer their injections (or for their caregiver to administer them), and making adjustments to the comfort settings of their eHealth device to minimize any pain upon injection, while ensuring that the correct dose of their treatment is delivered. Nurses also provide medical information aimed at parents and caregivers to encourage them to engage with the treatment to promote a successful outcome for their children.

Of the 120 patients identified as being in the “low adherence” group, 80 received an additional educational visit from a nurse at a time when the nurse would be in their locality to start another patient on r-hGH treatment. This visit to the non-adherent patients and their parents/caregivers was made between October 2015 and March 2016. At these visits (of ~1 h), the nurse investigated the particular cause(s) of low adherence in the individual patient and designed a personalized action plan: routines, reminders, and device adjustments, and provided the patients with specific information about adherence, missed injections, growth outcomes, etc. The data for this subgroup of patients were then retrospectively analyzed to compare adherence for the 6 months before and after the additional educational counseling (Figure 1).

Due to the observational nature of the study, it was not necessary to have ethics committee approval. As part of the PSP, all patients provided informed consent to enter the program and provide their anonymized data to be used for analysis.

### Statistical Analysis

Patient demographic data were tabulated and graphed. Statistical analyzes were carried out using STATA 15.0 software.

Continuous variables were presented as mean and median with their respective 95% confidence intervals (CIs), and categorical variables were presented as proportions with 95% CI. For an adherence comparison, the Wilcoxon signed-rank test was used. A multivariable linear regression was performed to assess which independent variables contributed to the variability of adherence in this population and *p* < 0.05 was considered significant. Potential confounders, including age, sex, and treatment duration were analyzed.

Because the distribution of the sample was non-parametric (non-normal distribution), the Wilcoxon signed-rank test was used for statistical comparison of paired groups.

## Results

Of the 80 patients in the low adherence group whose data were analyzed, 52 (65%) were male. Patients were aged 2–18 (mean: 11.77) years and duration of treatment was 0.40–11.13 (median: 3.62) years. The distribution of diagnoses is shown in Table 2. In terms of geographic distribution, the majority of the study population (91%) was located in five provinces within Argentina: Buenos Aires, Entre Rios, Córdoba, Tucumán, and Santa Fe (these data are consistent with the distribution of the population in Argentina).

**Table 2:** Distribution of diagnoses in the study population.

**Diagnosis**	**Frequency**	**Percentage**
Growth hormone deficiency	57	71.25
Chronic renal disease	1	1.25
Small for gestational age	16	20.00
Turner syndrome	6	7.50
**Total**	**80**	**100**

The adherence rates before and after the intervention showed that the nurse visit had a positive result, as median adherence was increased by 9% for three parameters: adherence to injections (number of injections vs. number of injections specified by the physician), dose (dose of r-hGH received vs. dose specified by the physician), and injection regimen (number of injections at the time specified by the physician vs. number of injections) (Table 3). The Wilcoxon signed-rank test showed statistically significant differences between the adherence values before and after the visit from the nurse (Table 4).

**Table 3:** Comparison of adherence before and after the educational intervention (nurse visit).

**Variable**	**Number of patients**	**Median**	**Q1**	**Q2**	**Min**.	**Max**.	**95% CI**	
Injection adherence (historical)	66	0.68	0.47	0.81	0.00	0.99	0.53	0.67
Injection adherence (baseline)	80	0.67	0.50	0.74	0.00	0.79	0.54	0.63
Injection adherence (after visit)	80	0.76	0.62	0.86	0.26	1.01	0.69	0.77
Dose adherence (historical)	66	0.69	0.41	0.81	0.00	1.00	0.54	0.68
Dose adherence (baseline)	80	0.67	0.48	0.74	0.00	1.05	0.55	0.64
Dose adherence (after visit)	80	0.76	0.62	0.86	0.26	1.01	0.68	0.76
Injection regime adherence (historical)	66	0.63	0.42	0.74	0.00	0.93	0.52	0.63
Injection regime adherence (baseline)	80	0.60	0.47	0.71	0.00	0.83	0.52	0.60
Injection regime adherence (after visit)	80	0.69	0.56	0.81	0.26	0.98	0.64	0.71
Age (years)	80	12.19	10.09	13.54	2.16	17.43	11.06	12.48
Time in treatment (years)	80	3.62	2.58	5.84	0.40	11.13	3.77	4.91

*CI, confidence interval*.

*Historical data = adherence data prior to baseline; Baseline data = 6 months prior to educational visit; Post-visit data = 6 months after visit*.

**Table 4:** Comparison between the paired groups: Median results of the Wilcoxon signed-rank test.

**Injection adherence (historical)**	**Injection adherence (baseline)**	**Injection adherence (after visit)**	***p***
0.68^*^“	0.67°”	0.76^*^°	^*^p **= 0.0252** **°*****p*** **= 0.0000** “*p* = 0.1676
**Dose adherence (historical)**	**Dose adherence (baseline)**	**Dose adherence (after visit)**	***p***
0.69^*^”	0.67°	0.76^*^°	^*^p **= 0.0331** **°*****p*** **= 0.0001** “*p* = 0.1117
**Injection regime adherence (historical)**	**Injection regime adherence (baseline)**	**Injection regime adherence (after visit)**	***p***
0.63^*^”	0.60°“	0.69^*^°	^*^p **= 0.0413** **°*****p*** **= 0.0002**” 0.0905

*Table compares the medians of the paired data in three time intervals: (1) historical data, (2) baseline data, and (3) post-educational intervention data and shows the results of the Wilcoxon signed-rank test that was performed*.

*Historical data = adherence data prior to baseline; Baseline data = 6 months prior to educational visit; Post-visit data = 6 months after visit*.

Further analysis was performed according to the historical adherence data recorded by the easypod™ device for these patients from the start of treatment. Statistically significant differences were found in the comparison between the historical values and the values after the visit (Table 4). Since adherence is a variable that could be modified over time, the analysis of historical data (adherence prior to baseline) allows us to assess whether there are differences between baseline and historical data and thus better understand the variations in the adherence in these patients.

**Figure 2:**
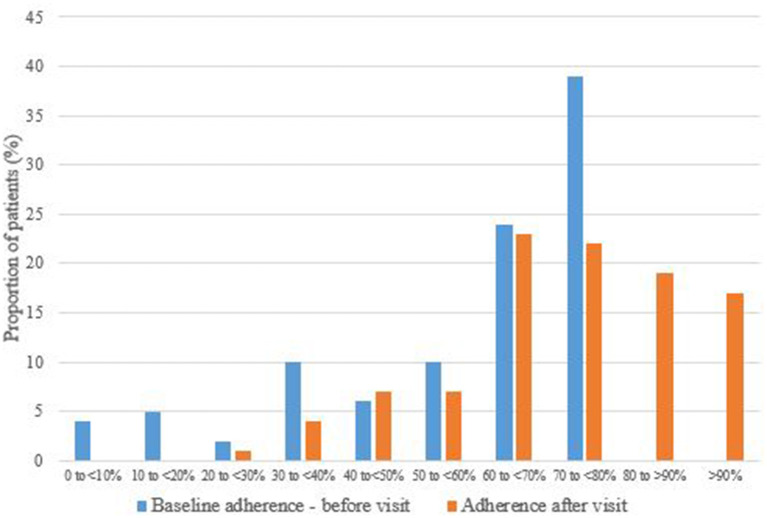
Change in injection adherence before and after the nurse visit.

A clearer way to interpret the results is to analyze how many patients changed from low to good adherence after the nurse visit. Of the 80 patients visited, 36.3% (29/80) increased their adherence to values considered as good adherence (≥80%) (Figure 2).

## Discussion

Low adherence to r-hGH treatment is common and is a substantial factor in cases of suboptimal growth outcomes. Interventions based on behavioral principles are effective in improving adherence, as demonstrated in other therapeutic areas (e.g., hypertension, AIDS, cancer, organ transplants, chronic asthma, diabetes, and obesity) (1). Our study demonstrates the value and effectiveness of the combination of innovative digital adherence monitoring devices and targeted educational interventions during the course of treatment in children with growth disorders.

This intervention was effective due to its multifactorial nature—the training and educational intervention was carried out by specialized healthcare professionals and an individualized approach was taken for each patient, according to their needs and the factors identified as involved in their low adherence. All patients were continuously monitored by the PSP nurses and call center staff. Conducting an educational visit in a home-based location was a convenient and accessible patient-oriented strategy and proved to be a successful, feasible, and replicable intervention with favorable clinical outcomes.

Although most research on interventions aimed at improving adherence has been focused on changing patient behavior, there is a growing trend toward targeting the physician and healthcare system through training and information sharing, in addition to targeting the patient. Moreover, there is some evidence that innovative, modified healthcare system teams, often including pharmacists, are more effective in addressing the adherence issue (6). The patients of physicians who share information and actively seek to improve their relationships through shared decision making with their patients also generally have better outcomes.

Research has also shown that no single intervention targeting patient behavior is fully effective, and the most promising methods of improving adherence behavior use a combination of patient education, behavioral skills, self-rewards, and social support. Simply following up by telephone can be one of the simplest and most cost-effective ways of improving adherence (1). Our nurse-led educational intervention was only one aspect of our PSP, and other components utilize different strategies such as behavior change techniques used by the PSP nurses and call center staff. Although some combinations of techniques have been shown to increase adherence and improve treatment outcomes, even the most effective do not appear to sustain their effects over the long term and few have been evaluated in randomized controlled trials to date (14)

### Strategies for Improving Adherence to Treatment With r-hGH

The implementation of strategies for the improvement of adherence to injectable therapy with r-hGH is associated with multiple clinical benefits, considering that it not only optimizes the effectiveness of treatment but also reduces unnecessary diagnostic and therapeutic efforts (3). Another important outcome of evaluating the adherence of patients treated with r-hGH is that it can help to differentiate between non-adherent patients and non-responders. In daily clinical practice it is often difficult to establish whether a poor response to treatment is secondary to lack of adherence or to an inadequate physiological response to the r-hGH administered (12, 15). The ability to exclude non-adherence to treatment in non-responders by electronic monitoring therefore represents an important advantage in patient care.

The recommended measures to promote adherence to treatment are directly related to the factors that contribute to poor adherence (3, 9). But first, it is essential to have methods capable of objectively evaluating the degree of therapeutic adherence of patients with r-hGH deficiency. As shown in our study, the advent of electronic devices designed to record the doses of r-hGH administered by the patient has provided physicians with the ability to observe real-time adherence to long-term treatment and identify patients who need help to optimize their adherence (5).

Another central strategy for the promotion of the therapeutic adherence of patients with GHD is education of the patient and their parent(s) in relation to the disease and the importance of treatment with r-hGH, not just on growth outcomes but on body composition, lipid metabolism, cardiovascular health, and improved quality-of-life (9). Physicians should evaluate whether there are any technical, physical, or psychological barriers that prevent optimal adherence being achieved by each individual patient, and should also consider the patient's lifestyle and the cost of treatment in their therapeutic decisions (3, 9).

Finally, improving the comfort of the subcutaneous injection of r-hGH represents another step forward for the improvement of therapeutic adherence in patients affected by GHD, as pain during injections is a common cause of poor adherence (16) (Table 1). The availability of digitally enhanced injection devices, like the easypod™, that allow the patient to adjust the speed of needle placement, infusion rate, and depth of injection according to their personal preferences, enables them to reduce pain and improve the comfort of subcutaneous administration (12).

We consider that the implementation of educational tools focused on strengthening the patient–physician relationship, and the availability of electronic devices that improve the comfort of r-hGH administration and objectively record adherence data, represent two effective strategies to optimize the benefits of treatment with r-hGH (3)

Use of the easypod™ device has been shown in a large multinational study (easypod™ connect observational study, or ECOS) involving 2,420 patients aged 2–18 years to help maintain adherence at high levels for up to 5 years (13). As sub-optimal adherence is associated with worse clinical and growth outcomes and with increased healthcare service utilization and greater healthcare spending (9), the combination of this device with a PSP should be cost-effective, even though it requires an investment in time by the PSP nurses and call center staff.

Limitations of the study include the relatively small number of patients involved, although our data were sufficiently robust to demonstrate statistical significance for the improvement in adherence after the visit from the nurse. Restricting inclusion in the study to low adherence patients living in the proximity of new patients who the nurses had to visit did limit the numbers involved, but made it possible to conduct the study in a realistic timeframe.

It is important to consider that there are currently no studies that effectively demonstrate how to improve adherence to r-hGH therapy, and that observational studies could be a first approach to generate a hypothesis for understanding what type of interventions could have a positive impact on adherence. Therefore, we suggest that further investigation by way of a randomized clinical trial, for example, is needed in order to confirm our findings.

## Conclusions

Sub-optimal adherence to r-hGH therapy is multifactorial and few methods have been proven fully effective in improving the long-term adherence required for optimal outcomes. However, based on our study, we conclude that a nurse-led educational intervention—as part of a PSP—is a simple method that improves adherence in patients with low adherence identified by electronic monitoring. We therefore recommend this type of intervention in these patients to reduce the gap between the indication/recommendation of the specialist and the patients' behavior.

## Data Availability Statement

Data sharing should be done in accordance with the European Federation of Pharmaceutical Industries and Associations (EFPIA) and the Pharmaceutical Research and Manufacturers of America's (PhRMA) Principles for Responsible Clinical Trial Data Sharing. Any requests for data by qualified scientific and medical researchers for legitimate research purposes will be subject to Merck KGaA's Data Sharing Policy. All requests should be submitted in writing to Merck KGaA's data sharing portal (https://www.merckgroup.com/en/research/our-approach-to-research-and-development/healthcare/clinical-trials/commitment-responsible-data-sharing.html). When Merck KGaA has a co-research, co-development, or co-marketing or co-promotion agreement, or when the product has been out-licensed, the responsibility for disclosure might be dependent on the agreement between parties. Under these circumstances, Merck KGaA will endeavor to gain agreement to share data in response to requests.

## Author Contributions

AA and AR designed the study and performed statistical analysis. FR extracted anonymized data and coordinated educational training. AA and CC discussed and interpreted the results. All authors provided critical feedback and helped shape the manuscript.

## Conflict of Interest

AA, FR, AR, and CC are all employed by Merck S.A. Argentina, Buenos Aires, Argentina, an affiliate of Merck KGaA, Darmstadt, Germany, and declare that this study received funding from Merck S.A. Argentina and from Merck KGaA, Darmstadt, Germany to support the submission for publication of this manuscript. The funder was involved in the study design, collection, analysis, or interpretation of data and funded additional medical writing support, as described in the Acknowledgments section.
